# Data of rational process optimization for the production of a full IgG and its Fab fragment from hybridoma cells

**DOI:** 10.1016/j.dib.2016.05.067

**Published:** 2016-06-02

**Authors:** Martina Röhm, Alina Handl, Maria König, Chrystelle Mavoungou, René Handrick, Katharina Schindowski

**Affiliations:** a Institute of Applied Biotechnology, University of Applied Sciences Biberach, Hubertus-Liebrecht-Strasse 35, 88400 Biberach, Germany; b University of Ulm, Faculty of Medicine, Albert-Einstein-Allee 11, 89081 Ulm, Germany

**Keywords:** Design of Experiment, Hybridoma, Papain, Production, Fab, F(ab′)_2_, Bioprocess

## Abstract

This data article focuses on the production of monoclonal antibodies (mAb) and their fragments Fab and F(ab′)_2_. Here, we present the data of an optimization protocol to improve the product yield of a hybridoma cell process using a Design of Experiment (DoE) strategy. Furthermore, the data of the evaluated conditions were used to test feeding strategies in shake flasks. They were verified in controlled 2 L fed-batch bioreactor processes. Supplementing the culture medium with human insulin-like growth factor-I (IGF-I) and Pluronic F-68, as well as a nutrient rich additive for fed-batch, resulted in improved cell growth correlating with a 7 day elongated process time and a 4.5 fold higher product titer. Finally, a rapid Fab generation protocol and the respective data are presented using different papain digestion and a camelid anti-kappa light chain VHH affinity ligand.

**Specifications Table**

**Table:** 

Subject area	*Biology*
More specific subject area	*Biotechnology/Process engineering*
Type of data	*Text file, graph, figure*
How data was acquired	*DoE using Modde software, different bioprocesses and analytical methods using microscopy, HPLC, SEC-MALS, SEC, SDS-PAGE and flow cytometry*
Data format	*Analyzed*
Experimental factors	*Sample were pretreated as indicated for each analysis*
Experimental features	*Process optimization for mAb production from hybridoma cells and generation of Fab/ F(ab′)2 fragment by papain digestion and a three step purification approach.*
Data source location	*Biberach/Riss, Germany*
Data accessibility	*Data is with this article*

**Value of the data**

• Our data provides a simple and fast optimization approach using Design of Experiment for media optimization in upstream processing.
• Antibody digestion revealed that parameters need to be adapted to each antibody subtype, which can result in various fragment yields.
• Our rapid and efficient protocol for Fab/F(ab′)_2_ generation can serve as a tool for other IgG digests and subsequent purification steps.

## 1 Data

The medium composition for a hybridoma cell line was optimized applying a simple Design of Experiment approach in shaking flasks, which was verified and provided the initial set-up for upstream processing. Based on the optimized medium, various feeding strategies with and without Cell Boost^TM^ 6 led to a prolonged process time and a higher mAb yield. For F(ab′)_2_/Fab generation, digestion parameters as well as affinity resins in downstream processing were evaluated for increased fragment yield coupled with SEC-MALS analysis and flow cytometry to confirm product quality and binding ability.

## 2 Experimental design, materials and methods

### 2.1 Hybridoma standard culture conditions

The mouse-mouse hybridoma cell line, producing the IgG_2a_ isotype anti-human insulin receptor mAb (AIR AB) 83-14, was grown in semi-adherent tissue culture flask with Dulbecco׳s modified Eagle׳s medium (DMEM, Biowest, Nuaillé, France) containing stable glutamine (4 mM) and glucose (4.5 g/L) supplemented with FBS (10% v/v) at 37 °C in a humidified atmosphere of 5% CO_2_. Under these conditions a product titer of 71.3 µg/mL was observed at day 4.

### 2.2 Media optimization using Design of Experiments

To improve productivity, three supplements – FBS (Sigma Aldrich, München/Germany), LONG®R^3^ IGF-I (Repligen, Waltham, MA, USA) and Pluronic® F-68 (Sigma Aldrich) - were used for optimization of the basal DMEM medium. Hence, Design of Experiment (DoE) was only applied for optimization due to known concentration ranges of IGF-I and Pluronic® from literature [3], [4], [5], which were used to reduce the amount of FBS.

**Fig. 1:**
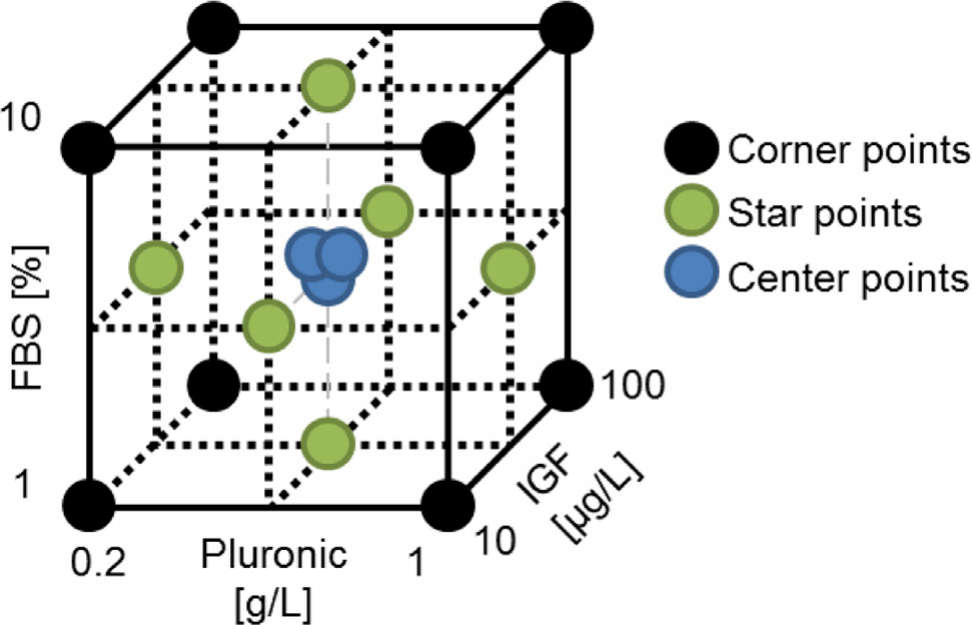
DoE Worksheet: Central composite face-centered design. Cube shows the distribution of the experiments for 3 supplements. The three center points describe the robustness and validity of the model.

The DoE cube shows the design space for the three supplements (Fig. 1) by using the Box Wilson central composite design (CCD) that includes factors, center points and star points to estimate the curvature [1]. Three concentration levels for each variable including a maximum (1), a minimum (−1) and a center point (0) were used as indicated in Table 1. The values shown in parenthesis are concentrations. Cells were seeded at 2×10^5^ cells/mL in 40 mL working volume in 125 mL disposable polycarbonate Erlenmeyer flasks (Corning, Steuben, NY, USA) in the respective culture medium for 3 days at 80 rpm, 37 °C (see Table 1).

Viability and viable cell concentration at day 3 were determined by trypan blue staining and quantification with an image-based cell counter (Cedex XS, Roche, Penzberg, Germany) as response factors and used for fitting and evaluating the model. The results were modeled with a polynomial equation to determine the effect of each factor and to predict the response of non-sampling points using a DoE software (Modde, Umetrics, Umea, Sweden) (see Supplementary data).

**Fig. 2:**
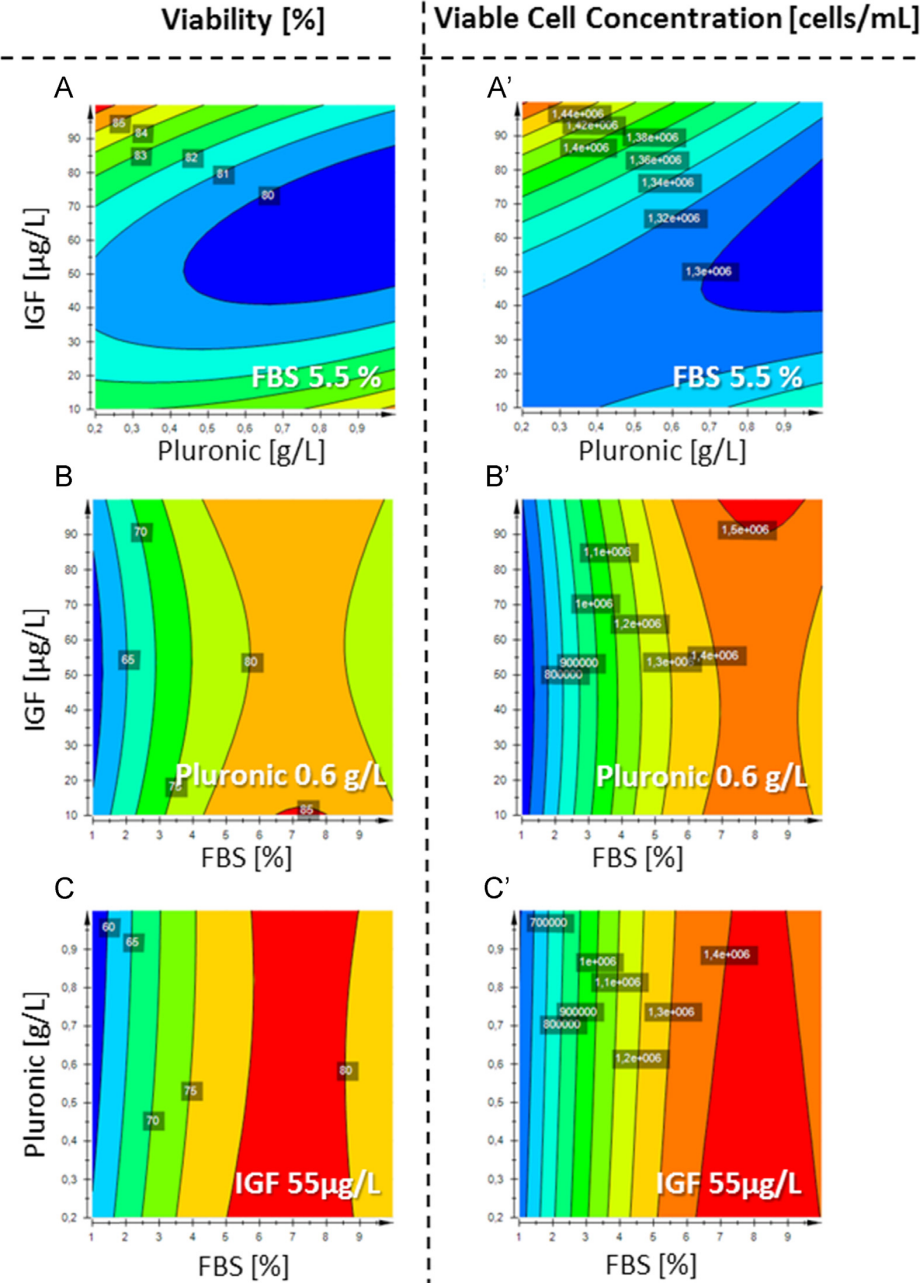
DoE data of medium optimization with supplements FBS, IGF-I and Pluronic® F68 regarding the effect on viability (A–C) and viable cell concentration (A′–C′). IGF-I and Pluronic® F68 were compared at constant FBS=5.5% (A and A′), IGF-I and FBS were compared at constant Pluronic®=0.6 g/L (B and B′) and Pluronic® and FBS were compared at constant IGF=55 µg/L (C and C′). Red color indicates the best and blue the worst conditions.

**Fig. 3:**
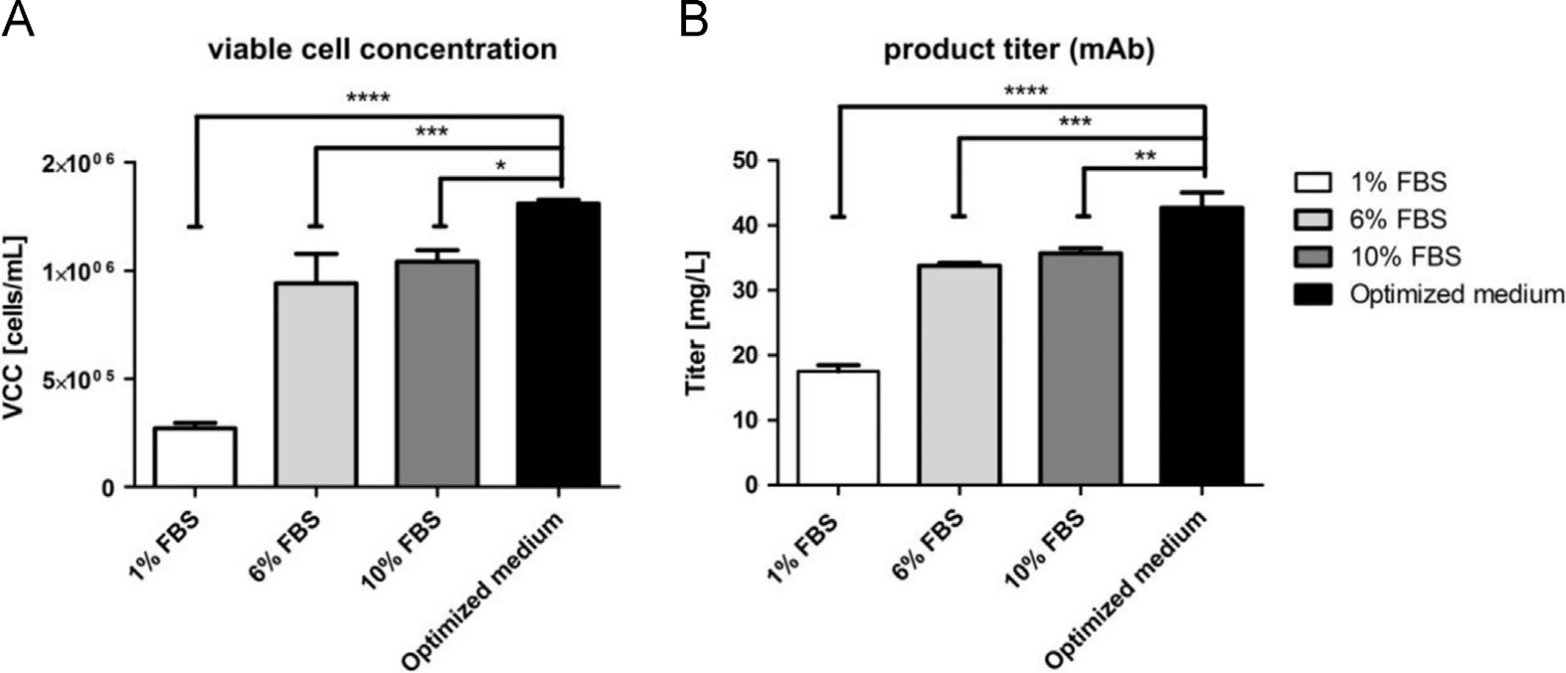
Data of DoE Validation: Viable cell concentration (A) and mAb titer (B) were analyzed in optimized inoculation medium (DMEM supplemented with 6% FBS, 100 µg/L IGF and 0.2 g/L Pluronic®) and controls (DMEM with 1, 6 and 10% FBS) (*n*=3, ±SD). At day 3 viable cell concentration and mAb titer were measured with an image-based cell counter (Cedex XS, Roche) and Protein A HPLC, respectively (*p<0.05, **p<0.01,***p<0.001, ****p<0.0001; one-way ANOVA).

Response contour blots revealed the predicted optimum, which was validated and compared to the original medium conditions in biological triplicates (*n*=3) and statistically evaluated in a one-way ANOVA (*p<0.05) (see Fig. 2, Fig. 3).

Based on the DoE data, FBS was decreased from 10% to 6% by supplementing the DMEM medium with 100 µg/L human IGF-I and 0.2 g/L Pluronic® F-68. Compared to the original culture conditions, an improvement in the cell growth rate by 9.7% (doubling time=24.5 h) was achieved within the exponential growth phase.

For validation, the optimized medium was compared to the original culture conditions and reduction of FBS from 10% to 6% and 1% without supplementation resulted in decreased viable cell concentration (−10% at 6% FBS and −74% at 1% FBS) and lower product titer (−5% at 6% FBS and −51% at 1% FBS). The optimized medium, compared to 10% FBS supplementation, showed a significant impact on viable cell concentration (Fig. 3A) and product titer (Fig. 3B) by 26.1% (±8.1%) and 19.7% (±4.8%), respectively.

### 2.3 Fed-batch feeding strategy

Optimized growth medium as basal medium was used to establish a fed-batch bioprocess. Seeding was done in 35 mL optimized medium (DMEM, 4.5 g/L glucose, 2 mM stable glutamine, 6% FBS, 100 μg/L IGF-I and 0.2 g/L Pluronic® F-68) using 3×10^5^ cells/mL in shake flasks (125 mL). For fed-batch feeding Cell Boost^TM^ 6 (CB6, Hyclone^TM^, GE Healthcare, Birmingham, UK), an alternative to a serum-free conventional glucose feed, was used that contains glucose, vitamins, trace elements, amino acids, growth factors, lipids and cholesterol. Glutamine and glucose were kept constant at 2 g/L and 1 mM, respectively. The first triplicate served as batch control and was cultivated without feeding. The next triplicate received 20 mM glutamine and 20 g/L glucose. Instead of usual glucose feed in the medium the third triplicate was fed with 14 g/L glucose in CB6. All substrates and metabolites were determined with a Konelab^TM^ Arena 20XT (Thermo Scientific, Rockford, IL, USA), the cell concentration was analyzed by an image-based cell counter (Cedex XS, Roche, Penzberg, Germany) and the mAb concentration (product titer) was evaluated with a Protein A HPLC (Agilent).

**Fig. 4:**
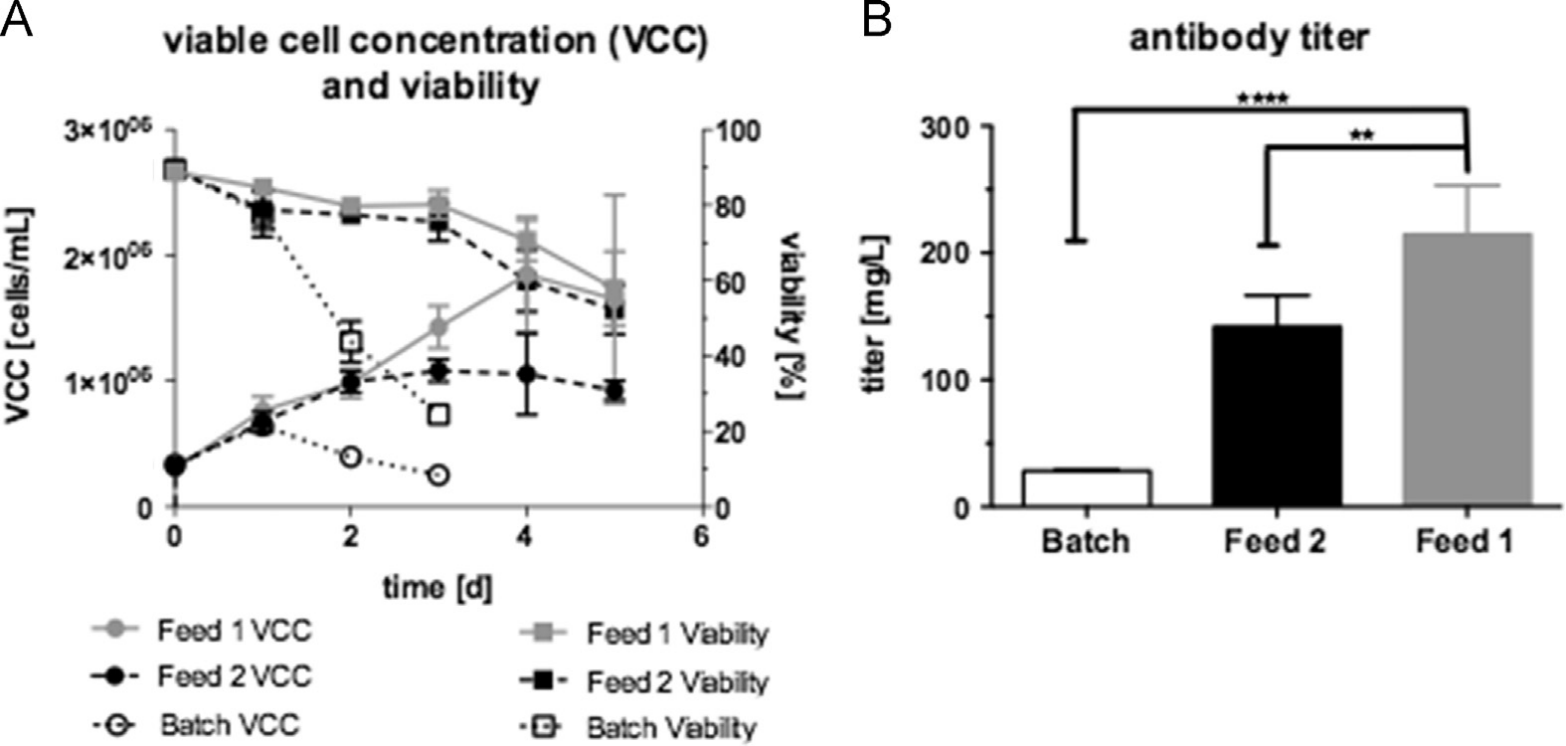
Data of double bolus feed: Feed 1 contained Cell Boost^TM^ 6 (CB6) (3.5% w/v) supplemented with glucose and Feed 2 w/o CB6 contained glucose in optimized medium. (B) mAb titers are depicted from the last day of culture (*p<0.05,***p<0.001; one-way ANOVA).

The data obtained revealed that CB6 improved cell growth, cell viability (Fig. 4A) and product (mAb) titer (Fig. 4B). The use of CB6 (Feed 1) increased the viable cell concentration at day 4 by 18.4% compared to conventional feed (Feed 2). As the viable cell concentration correlates with the product titer, the mAb concentration peaked at 199.7±43.5 mg/L using Feed 1 compared to Feed 2 (127.3±2.1 mg/L), an increase of over 36%.

**Fig. 5:**
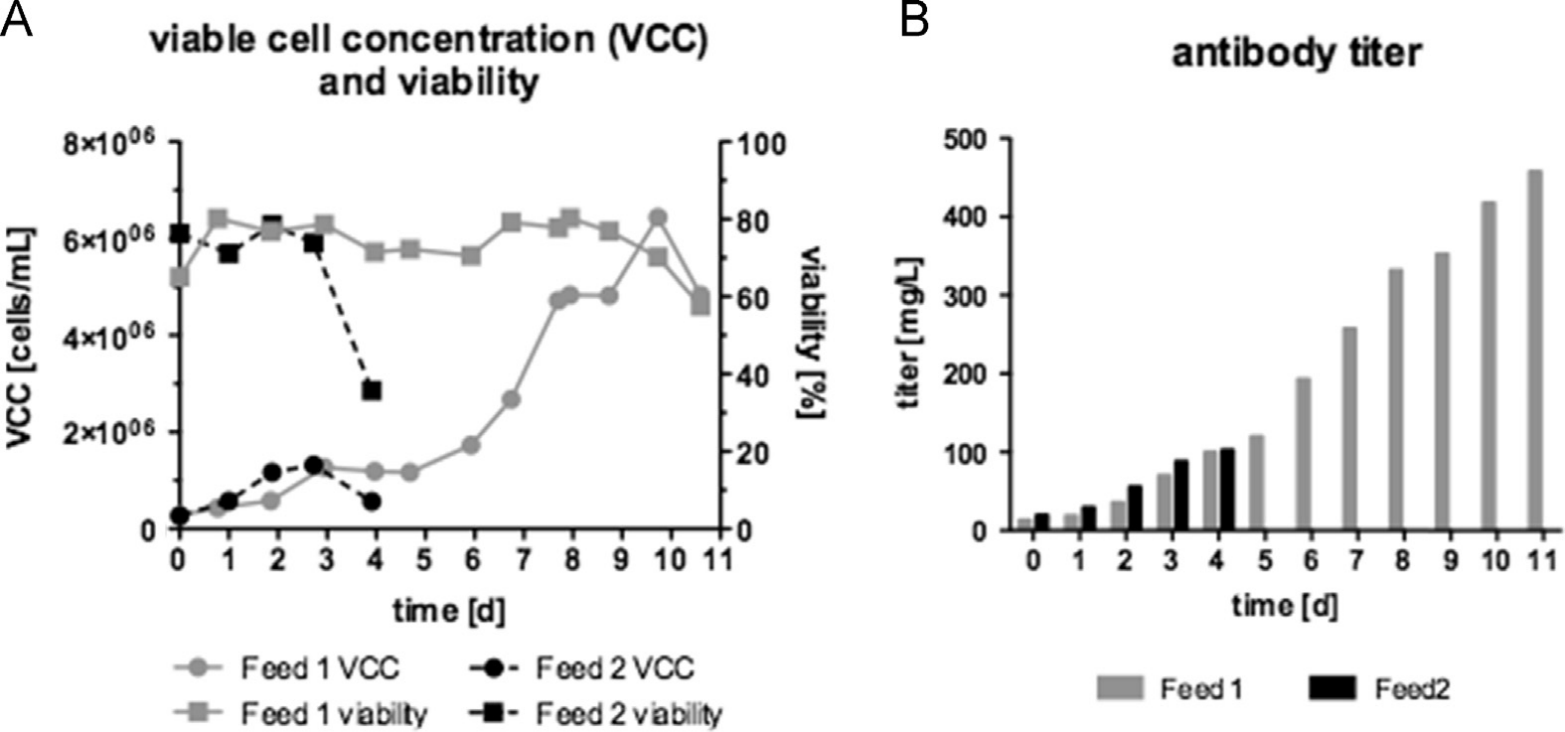
Data of Fed-batch 2 L process with double feed – glutamine in medium and glucose (Feed 1: with CB6; Feed 2: w/o CB6). Fed-batch was started with optimized medium (DMEM supplemented with 6 % FBS, 100 µg/L IGF and 0.2 g/L Pluronic®).

Both feeding strategies with and without CB6 were evaluated in a 2 L fed-batch bioprocess. Under controlled conditions the CB6 feed revealed a strong benefit allowing a prolongation of the process time from 4 to 11 days (Fig. 5A) and a 4.5-fold higher product titer of 458 mg/L compared to 102 mg/L (Fig. 5B).

### 2.4 Capturing of full size antibody and generation of Fab and F(ab′)_2_

**Fig. 6:**
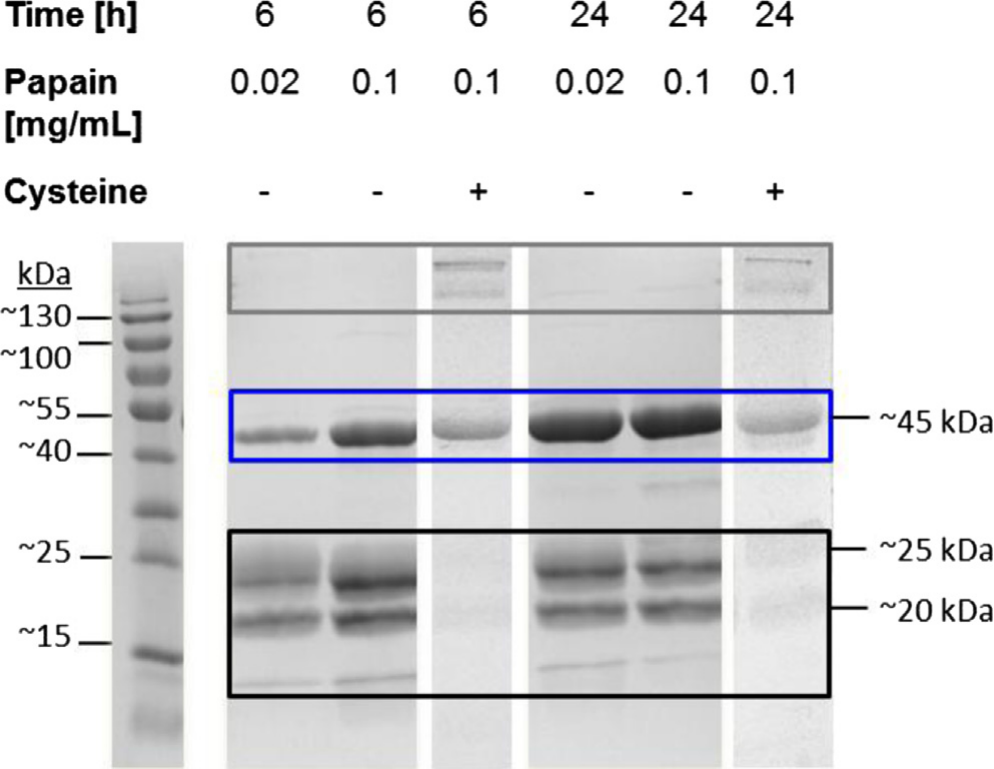
Data of different papain concentrations and incubation period. The effect with and without cysteine (+, −) on mAb fragmentation was analyzed with constant papain and mAb concentration at different incubation times. Results were analyzed by 12.5% SDS-PAGE under non-reducing conditions and stained with Coomassie brilliant blue. Box: upper=undigested mAb; middle=Fab fragment; lower=Fc fragment, degraded mAb.

Subsequent to cell culture, the supernatant was microfiltered, AIR AB 83-14 IgG was captured by Protein A affinity chromatography (MabSelect® SuRe resin, Äkta Purifier, GE Healthcare) and eluted at 50 mM sodium acetate (pH 3.5). Papain (Sigma Aldrich) was pre-activated with cysteine in activation buffer (0.02 M cysteine, 0.02 M EDTA in PBS) and dialyzed against digestion buffer (1 mM EDTA in 50 mM sodium phosphate pH 6.3) in order to remove cysteine. Different papain concentrations (see Fig. 6) and incubation periods were used, with and without the presence of cysteine for Fab fragmentation. The mAb (2 mg/mL) was diluted in papain digestion buffer 1:1, incubated at 37 °C at 200 rpm agitation and reaction was terminated by a final iodoacetamide concentration (0.03 M) (Sigma Aldrich). Incubation over 24 h of the full IgG in the presence of 0.1 mg/mL papain resulted in the largest amount of Fab (Fig. 6).

**Fig. 7:**
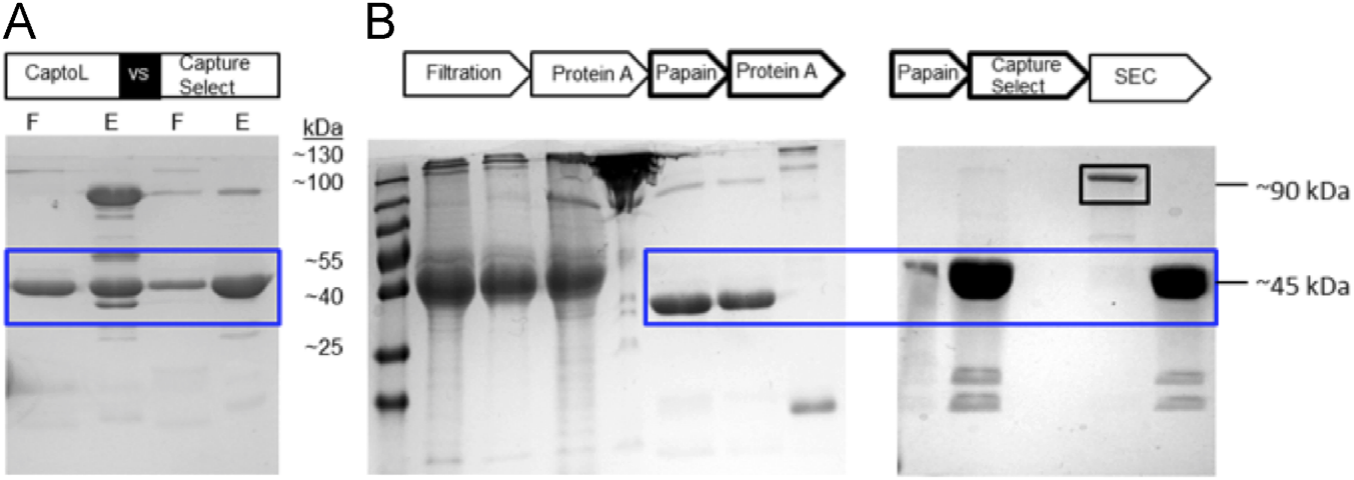
Data of purification using two affinity anti-kappa light chain resins, CaptoL and CaptureSelect, for Fab polishing (A) and the complete Fab purification process (B). In B, the second Protein A step was replaced by CaptureSelect after papain digestion. Results were analyzed by 12.5 % SDS-PAGE under non-reducing conditions and gels were stained with Coomassie brilliant blue. Blue box=Fab fragment, F=Flow through and E=Elution.

Subsequently, the influence of cysteine needed to pre-activate papain at the lowest destabilizing effect on the IgG domains was determined by semi-quantitative SDS-PAGE where equal amounts were loaded. Therefore, papain was activated with cysteine (20 mM) in activation buffer and dialyzed against digestion buffer to remove cysteine. The mAb digestion without cysteine showed less undesirable product related fragments between 20 and 25 kDa and increased Fab yield (Fig. 6). Furthermore, this digestion protocol resulted in the production of F(ab′)_2_ fragments (Fig. 7).

The solution was dialyzed against PBS for further purification. Two anti-kappa light chain resins – the Protein L based CaptoL (GE Healthcare) and the VHH resin CaptureSelect^TM^ LC-kappa (mur) (Life Technologies, Carlsbad, CA, USA) – were evaluated with the same equilibration and elution buffer (PBS pH 7 and 20 mM sodium acetate pH 3.5). VHH chromatography resulted in a highly specific Fab binding and less by-products were found in the elution fraction compared to the Protein L resin (Fig. 7A). Due to the high specificity of the camelid VHH resin, the second Protein A step was dispensable. F(ab′)_2_ and Fab were successfully separated by a subsequent size exclusion chromatography (SEC; HiLoad 16/60 Superdex 75, GE Healthcare) as shown in SDS-PAGE analysis (Fig. 7B). Based on the bioreactor supernatant as 100% full IgG, the yield calculation resulted in approximately ~38% Fab at the end of the purification process.

### 2.5 Product identity and bioactivity as quality control

The state of aggregation and purity of resulting Fab, F(ab′)2 and full IgG were analyzed after Protein A, VHH chromatography and SEC purification step via SEC-Multi-angle light scattering (MALS). Purified products were dissolved in PBS (pH 7.2 (Sigma Aldrich)) and loaded on a SEC column (Yarra SEC-3000 and SEC-2000, Phenomenex, Aschaffenburg, Germany) for molecular separation of all components using a flow of 0.5 mL/min.

**Fig. 8:**
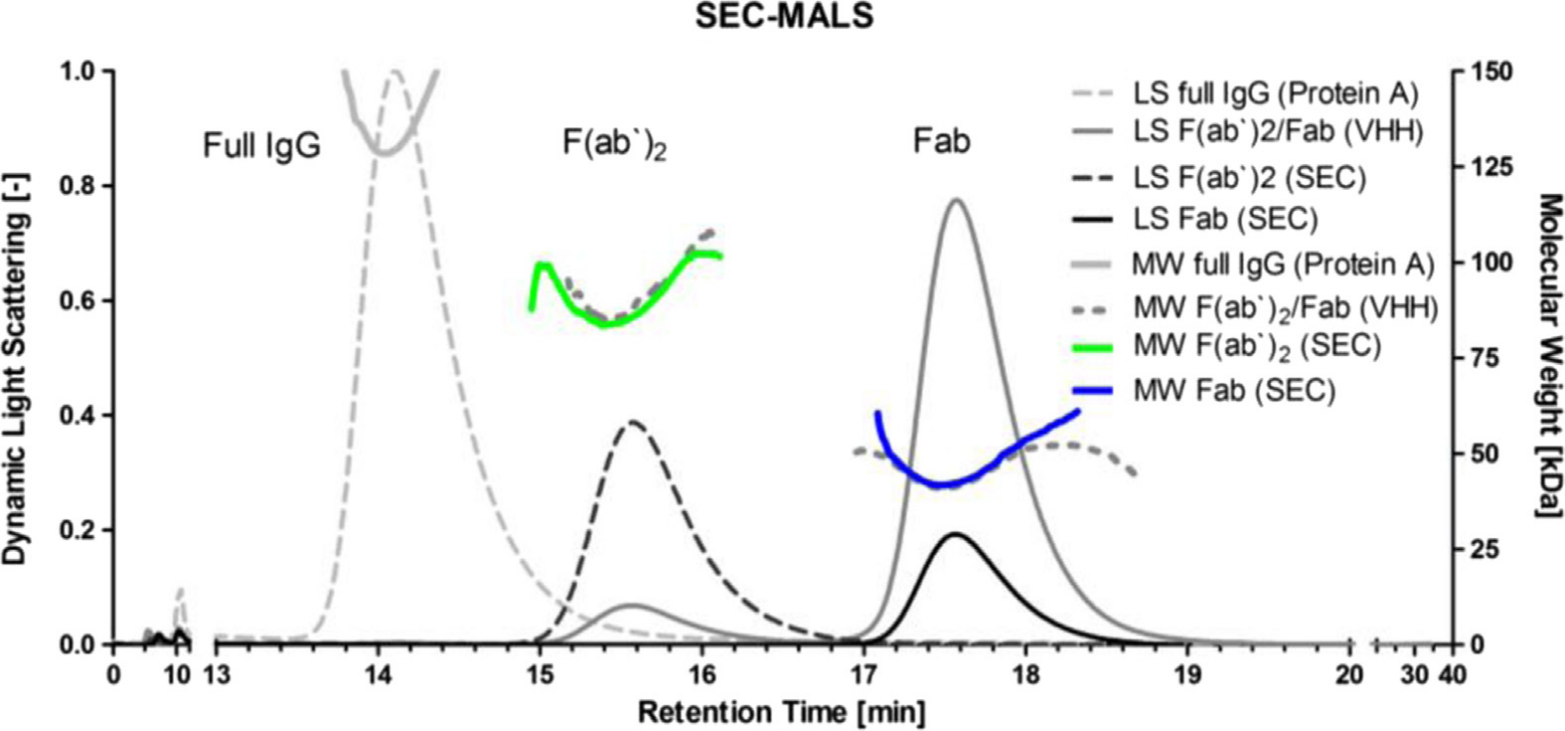
SEC-MALS data of purified full IgG (Protein A), F(ab′)_2_ /Fab (VHH) and F(ab′)_2_ and Fab (both SEC). Dynamic Light Scattering (LS) signals are shown as peaks in gray shades and the molecular weight as horizontal lines. The MW of purified F(ab′)_2_ and Fab is indicated as green and blue colored line.

Peaks were detected by UV (280 nm), refractive index and MALS detector (Wyatt, Santa Barbara, USA) for determination of molecular weight and aggregation. The full IgG had a molecular weight of 143.9±1.9 kDa. Subsequent to VHH column elution, two signals were detected – one with 91.5±2.2 kDa and one with 44.73±0.5 kDa correlating to the predicted size of the corresponding F(ab′)_2_ and the Fab fragment. Finally, Fab was successfully separated from F(ab′)_2_ by size exclusion chromatography as confirmed by SDS-PAGE analysis (Fig. 7) and by SEC-MALS with a molecular weight of 45.3±0.5 kDa and 90.6±1.0 kDa, respectively (Fig. 8).

**Fig. 9:**
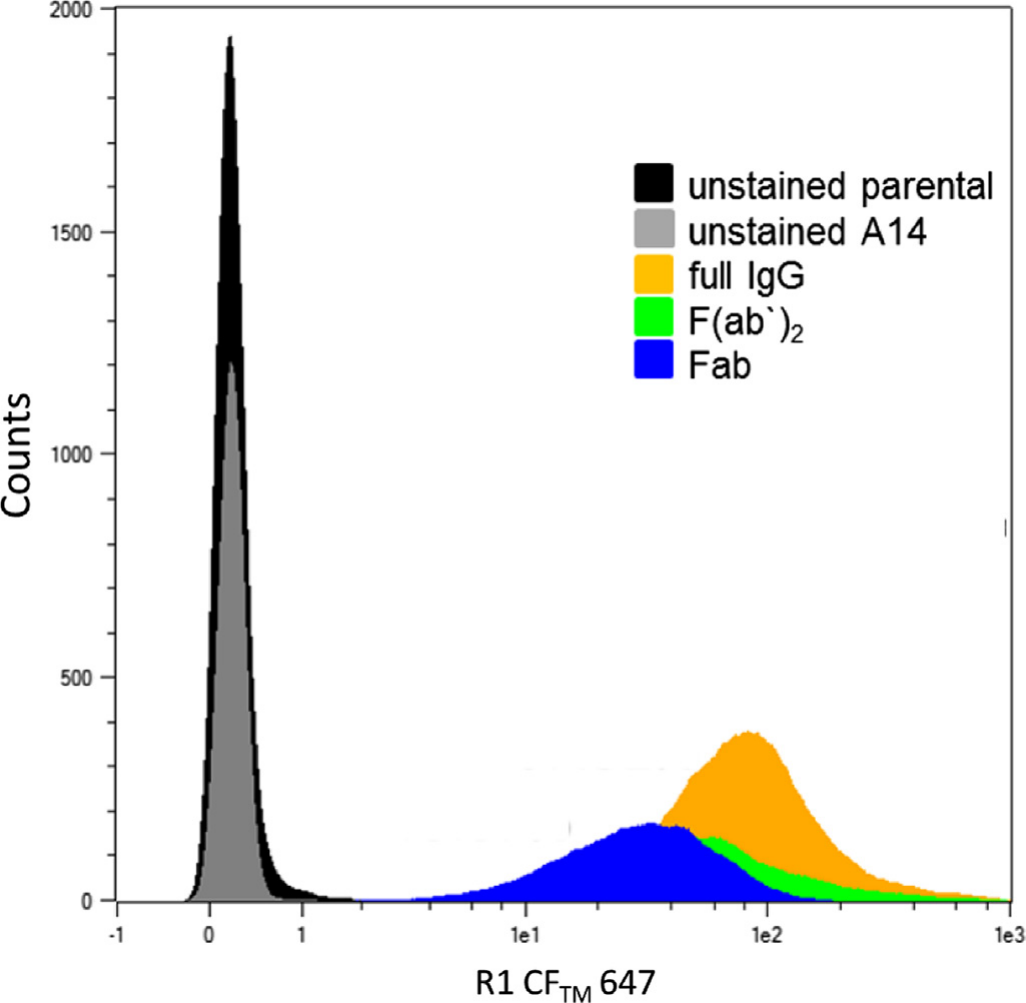
Data of antigen-binding: Biding of labeled products to antigen-expressing A14-NIH3T3 cells were analyzed by flow cytometry. Non-expressing parental NIH3T3 cells and unstained A14 were used as negative control. Only viable cells were gated.

To analyze the antigen binding of the products Fab, F(ab′)_2_ and full IgG were directly labeled with equal amounts of the fluorophore CFTM 647 (Mix-n-Stain, Sigma Aldrich) according to the manufacturer׳s instruction. The labeled products were used for extracellular staining of mouse fibroblast NIH3T3-A14 cells expressing the antigen, human insulin receptor [2]. For flow cytometry analysis, cells were stained with fluorophore labeled mAb or fragments (2 µg/mL) for 30 min at room temperature in the dark prior to data acquisition using a MACSQuant flow cytometer (Miltenyi Biotec GmbH, Bergisch Gladbach, Germany). The parental cell line NIH3T3 was chosen as negative control and compared to the antigen expressing A14 cells. All labeled proteins resulted in a shift of the fluorescence detection channel according to their theoretical number of coupled fluorophores that is correlated to their molecular mass (Fig. 9). The binding ability of the mAb and its fragments to their antigen was preserved during downstream processing and papain digest.

## Figures and Tables

**Table 1:** Data of DoE factors and responses at day 3: The lowest concentration is indicated as −1, the mid concentration as 0 and the highest with +1. The values shown in parenthesis are the concentrations. Expt 18–24 are additional controls.

Expt	Factor variables			Response variables	
	FBS [%]	Pluronic® [g/L]	IGF [µg/L)	Viability [%]	Viable cell concentration [10^6^ cells/mL]
1	−1 (1)	−1 (0.2)	−1 (10)	75.8	0.74
2	1 (10)	−1 (0.2)	−1 (10)	79.1	1.47
3	−1 (1)	1 (1)	−1 (10)	66.6	0.58
4	1 (10)	1 (1)	−1 (10)	82.0	1.40
5	−1 (1)	−1 (0.2)	1 (100)	71.6	0.70
6	1 (10)	−1 (0.2)	1 (100)	77.9	1.55
7	−1 (1)	1 (1)	1 (100)	59.3	0.55
8	1 (10)	1 (1)	1 (100)	78.7	1.32
9	−1 (1)	0 (0.6)	0 (55)	69.1	0.63
10	1 (10)	0 (0.6)	0 (55)	79.8	1.46
11	0 (5.5)	−1 (0.2)	0 (55)	82.5	1.46
12	0 (5.5)	1 (1)	0 (55)	78.9	1.44
13	0 (5.5)	0 (0.6)	−1 (10)	79.1	1.33
14	0 (5.5)	0 (0.6)	1 (100)	81.6	1.34
15	0 (5.5)	0 (0.6)	0 (55)	81.8	1.27
16	0 (5.5)	0 (0.6)	0 (55)	80.2	1.19
17	0 (5.5)	0 (0.6)	0 (55)	81.3	1.19
18	0	0.6	55	28.5	0.01
19	5.5	0	55	78.4	1.26
20	5.5	0.6	0	81.3	1.27
21	5.5	0.2	100	83.7	1.53
22	10	0	0	83.6	1.28
23	6	0	0	81.9	1.31
24	1	0	0	57.2	0.47
