# Supplementary material for: Data of rational process optimization for the production of a full IgG and its Fab fragment from hybridoma cells
Source: Data Brief. 2016 Jun 2;8:426–35. doi: 10.1016/j.dib.2016.05.067 (PMC4910301; doi:10.1016/j.dib.2016.05.067)
Supplement: Supplementary file 1 — Supplementary material [file mmc1.pdf]

AUTHOR DECLARATION (Data in Brief)

We wish to confirm that there are no known conflicts of interest associated with this publication and there has been no significant financial support for this work that could have influenced its outcome.

We confirm that the manuscript has been read and approved by all named authors and that there are no other persons who satisfied the criteria for authorship but are not listed. We further confirm that the order of authors listed in the manuscript has been approved by all of us.

We confirm that we have given due consideration to the protection of intellectual property associated with this work and that there are no impediments to publication, including the timing of publication, with respect to intellectual property. In so doing we confirm that we have followed the regulations of our institutions concerning intellectual property.

We understand that the Corresponding Author Dr. Katharina Schindowski Zimmermann is the sole contact for the Editorial process. She is responsible for communicating with the other authors about progress, submissions of revisions and final approval of proofs. We confirm that we have provided a current, correct email address (zimmermann@hochschule-bc.de) which is accessible by the Corresponding Author and which has been configured to accept email from

Signed by all authors as follows:

Martina Stützle 8.1.2016
Martina Stützle (signature and date)

Chrystelle Mavoungou (signature and date)

A. Handl 8.1.16
Alina Handl (signature and date)

Handrick, 2016-01-09
René Handrick (signature and date)

M. König 12.01.16
Maria König (signature and date)

Prof. Dr. Katharina Zimmermann
Molekulare Pharmakologie & Biochemie
Fakultät Biotechnologie
Hochschule Biberach
Hubertus-Liebrecht-Str. 35
D-88400 Biberach/Riss
HBC. HOCHSCHULE BIBERACH UNIVERSITY
30th Dec 2015
Katharina Schindowski Zimermann (signature and date)
